# Making hydroxyurea affordable for sickle cell disease in Tanzania is essential (HASTE): How to meet major health needs at a reasonable cost

**DOI:** 10.1002/ajh.26007

**Published:** 2020-10-06

**Authors:** Enrico Costa, Prosper Tibalinda, Enrico Sterzi, Hubert M. G. Leufkens, Julie Makani, Eliangiringa Kaale, Lucio Luzzatto

**Affiliations:** ^1^ Department of Pharmacy Azienda Ospedaliera Universitaria Integrata Verona Verona Italy; ^2^ Utrecht WHO Collaborating Centre for Pharmaceutical Policy and Regulation Utrecht The Netherlands; ^3^ School of Pharmacy Muhimbili University of Health and Allied Sciences Dar‐es‐Salaam Tanzania; ^4^ Department of Haematology Muhimbili University of Health and Allied Sciences Dar‐es‐Salaam Tanzania

1

To the Editor:

More than 300 000 babies with sickle cell disease are born every year worldwide, and more than three‐quarters are in Africa. Tanzania is among the five countries in the world that rank highest with respect to this burden. In view of this, in 2004 the Muhimbili University of Health and Allied Sciences (MUHAS) and the Muhimbili National Hospital (MNH) established the Muhimbili Sickle Cell (MSC) program, a systematic and comprehensive program aimed to integrate research, training, education and advocacy into the care of SCD in the country.^1^

Definitive cure of SCD can be achieved only by bone marrow transplantation, a procedure not free of risk that requires ad hoc facilities and is currently out of reach for most patients living in Africa; or by gene therapy that holds great promise, but must be regarded as being still at an experimental stage. Recently new drugs have been approved in high income countries (glutamine, voxelotor, crizanlizumab), but their role in the management of SCD patients at large is still to be defined.^2^

At the moment, the one medicine that has been universally established as part of the standard of care for SCD is hydroxyurea (HU). In view of its proven efficacy and long‐term safety HU was listed by the World Health Organization (WHO) as an essential medicine; and in a formal trial it has been shown to be valuable, as expected, in Africa as it is elsewhere^3^; however, the sad reality is that the majority of patients in this continent are in fact not receiving this medicine.

In the aim to correct this serious shortcoming in the management of SCD, we first wished to assess the availability and affordability of HU in Dar‐es‐Salaam, Tanzania. The collaborative partnership between WHO and the international non‐governmental organization Health Action International (WHO/HAI) developed a methodology for measuring the availability and affordability of medicines worldwide. The WHO/HAI defines a drug as *available* when found in a particular pharmacy on the day of data collection; as *affordable* when the out‐of‐pocket cost of a one‐month course of treatment is less than 1 day salary of the lowest‐paid unskilled government worker (LPUGW); and as *accessible* when it is at the same time available and affordable.^4^ In two surveys (carried out in 2016 and in 2018), we found that HU was not available in three out of five pharmacies (Figure 1A). From interviews with pharmacists we learned that HU was not in stock because level of demand was low. The selling price ranged from USD 0.27 per 500 mg capsule in the MNH Hospital Pharmacy, to USD 0.91 in one of the nearby retail pharmacies. Therefore the average cost for a monthly course of treatment (1 g/day for 30 days) was USD 35.16 (range: 16.50‐54.60 USD). Since in Tanzania the estimated daily salary of the LPUGW is USD 1.4,^5^ to procure 1 month of HU treatment would require on average 25.11 days, rather than one (Figure 1A). Thus, by WHO/HAI definition, HU was poorly available, not affordable, and therefore not accessible. We also note that the prices in the pharmacies visited were between 1.2 and 4.1 times higher than the international reference price (IRP: USD 0.22 per capsule in 2018).

**FIGURE 1:**
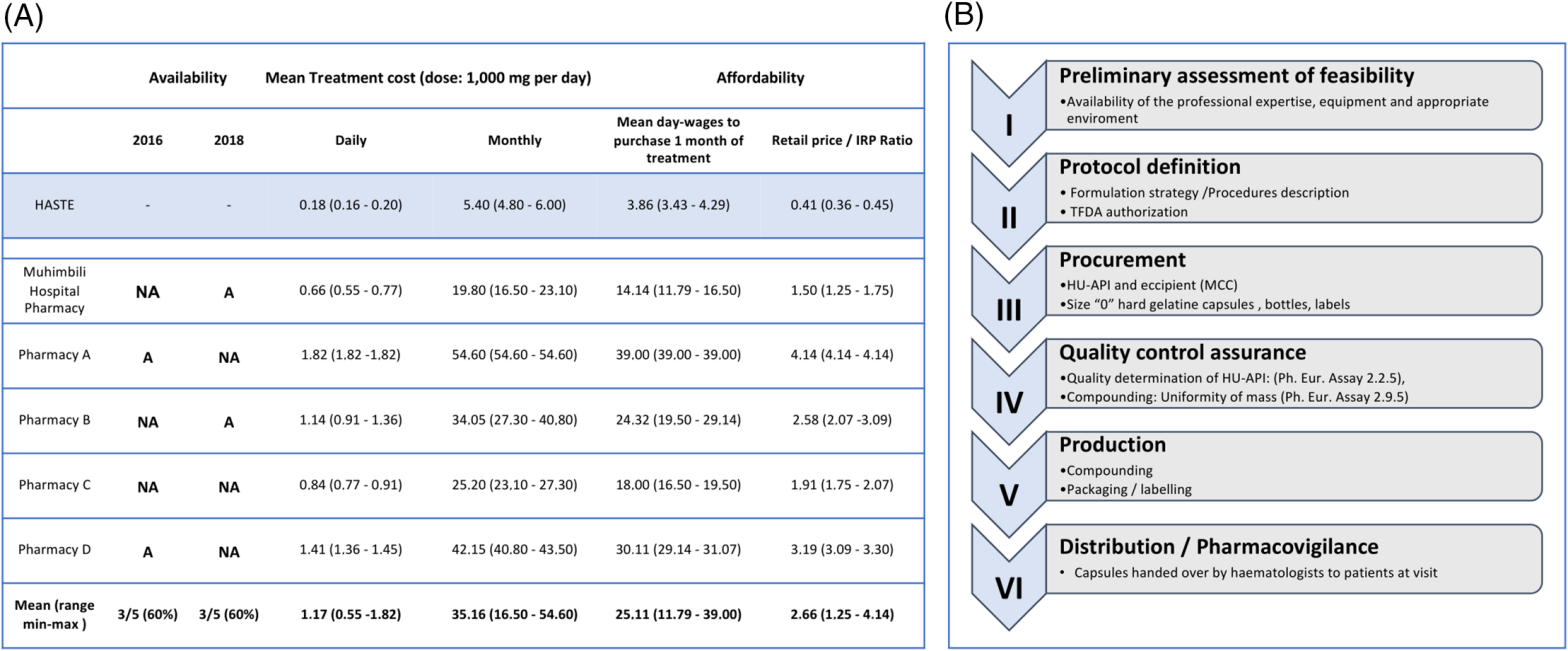
Curbing the price of HU to SCD patients by offering a galenic preparation. A, Availability, prices and affordability of HU in Dar es Salaam. A, Available; NA, Not Available. The price at which HU would have sold, whether available or not on the day of the interview, is expressed in USD (average exchange rate: 1 USD = 2200 Tanzanian shillings ‐ TZS). The Median price ratio (MPR) ‐ the price of HU from the survey divided by the international reference price (IRP) ‐ was used to contextualize local prices at international level. B, Stepwise approach to the compounding process for hydroxyurea at MUHAS

Next, we explored pharmacy compounding as a modality that might make HU more accessible to SCD patients in Tanzania. Compounding is the act of preparing, mixing, assembling, packaging, and labelling a drug in response to a practitioner's prescription drug order or initiative based on the practitioner‐patient‐pharmacist relationship in the course of professional practice.^6^ We recalled suggestions made one century ago by Robert P Fischelis: he pointed out that in certain cases health care professionals can use their own know‐how efficiently, and he exhorted the “pharmacist to utilize all means at his command in an effort to meet the demands of physicians, and at the same time supply the remedies prescribed at as reasonable a price as possible”.^7^ We took heed from this seminal paper, which was published at about the same time that James B. Henrick reported the first case of SCD. Since the HU patent expired a long time ago, there is no direct legal impediment to the galenic trajectory.

As a first step, we obtained permission from the Tanzania Food and Drug Authority (TFDA) to administer compounded HU to named SCD patients at the Hematology Clinic of MNH. Next we purchased raw materials, that is, HU Active Pharmaceutical Ingredient, microcrystalline cellulose excipient, and hard gelatine capsules, from which, at the School of Pharmacy, MUHAS. Compounding of HU was carried out in compliance with international Pharmacopeia standards, including quality controls by high performance liquid chromatography through the comparison of the purchased HU API with the standard obtained from the European Directorate for the Quality of Medicines & Healthcare (EDQM), (Figure 1B). Finally, HU capsules were dispensed directly to 41 adult patients during clinic visits, providing each patient with a supply of HU sufficient for 1.5 months. We had no reason to carry out a formal trial; however, we have reviewed data from clinic visits on 26 out of 41 patients. Before galenic HU the mean hemoglobin level was 7.10 g/dL (SD 1.55; range 3.2‐9.6), and the mean MCV was 88.2 fL (SD 6.36; range 73‐102). After galenic HU the mean hemoglobin level was 7.93 g/dL (SD 1.50; range 3.7‐10.1), and the mean MCV was 97.5 fL (SD 8.44; range 80‐115). In 11 out of 26 patients the Hb increment was greater than 1 g/dL.

In this pilot experiment HU was issued free of charge to the patients, thanks to a donation from an outside source and to the good will of all MUHAS staff concerned. Given that we have established practical feasibility of this approach, we proceeded to work out a budget required to produce a 1‐year HU supply for the treatment of 1000 patients ‐ such as may be needed for a cohort of SCD patients at a referral center in Africa. We estimate the total cost at USD 66 613, of which paid personnel would be 40%, raw materials 27%, amortization of equipment 13%, add‐on costs (import permits, energy, supplies, etc.) 20%. By this estimate, the final cost per daily dose of 1000 mg would be USD 0.18. Thus, compared with the products from our survey, pharmacy compounded HU from HASTE would be 3.6 times cheaper than commercially available HU from the hospital pharmacy, and 5 to 10 times cheaper than HU from the retail market in Tanzania (Figure 1A).

When a person in Tanzania has an acute illness, for example, from bacterial infection, that person and family will make sacrifices to buy a course of an antibiotic ‐ even if not affordable by the WHO/HAI definition. The situation is obviously quite different with a chronic, lifelong disorder like SCD. Given a poverty rate in Tanzania of 26.8%, many patients would be unable to purchase HU for a lifetime.

Over the past several decades attempts have been made to negotiate lower prices of drugs through tendering or purchasing agreements, but these have not been very successful. In principle, access to health care can be provided in one of two ways. On the one hand, the so‐called *vertical programmes* provide medicines for specific diseases from international agencies. Unfortunately, SCD, although recognized as a major public health issue in Sub‐Saharan Africa, has not yet received from international donors the funding that has accrued to HIV, TB or malaria: we think this anomaly should be corrected.^8^ On the other hand, horizontal programs are aimed at improving the capacity of the pharmaceutical system as a whole, and ultimately to provide universal health care (as in Rwanda).^9^

In conclusion, our test setting of pharmacy compounded HU has proven to be economically advantageous with respect to this essential medicine. The TFDA regulatory approval for a named patient approach has been essential; and the tasks of ensuring compounding capacity, quality control management, and trust building among prescribers, pharmacists and patients have passed the test. However, it is clear that, in the long run, pharmacy compounding cannot be a long‐term solution. Ultimately, for the SCD patient population to have full benefit from HU will depend on policy and political decisions by individual governments. We hope that this pilot study may encourage drug developers, manufacturers and indigenous entrepreneurs to invest in African countries, especially where necessary local expertise is already present. This is also in line with the recommendations of the UN High‐Level Panel on Access to Medicines,^10^ recommending strengthening of manufacturing capacity as a measure to improve accessibility to essential medicines.

## 2 CONFLICT OF INTEREST

The authors declare no conflict of interest.

## AUTHOR CONTRIBUTIONS

E.C. conceived of the study, participated in its design, carried out the survey and the budget impact analysis and wrote the paper. P.T. performed the compounding. E.S. participated in the design of the study. H.L. participated in the planning of analyses and interpretation of results. J.M. participated in the coordination of the study, and in the recruitment and follow up of patients. E.K. conceived of the study, participated in its design and performed the quality control analysis. L.L. conceived of the study, participated in its design, carried out the survey and the budget impact analysis and wrote the paper.

All authors helped with the manuscript: they read and approved the final version.

## FUNDING INFORMATION

The charity Associazione Piera Cutino in Palermo (Italy) funded the purchasing of the raw materials for the compounding of hydroxyurea, and A.C.E.F. LTD supplied MCC for free.

3

## Data Availability

The data that support the findings of this study are available from the corresponding author upon reasonable request.
